# Idiosyncratic and shared contributions shape impressions from voices and faces

**DOI:** 10.1016/j.cognition.2024.105881

**Published:** 2024-07-18

**Authors:** Nadine Lavan, Clare A.M. Sutherland

**Affiliations:** a Department of Biological and Experimental Psychology, School of Biological and Behavioural Sciences, Queen Mary University of London, United Kingdom; b School of Psychology, King’s College, University of Aberdeen, United Kingdom; c School of Psychological Science, University of Western Australia, Australia

**Keywords:** Voice, Face, First impressions, Individual differences, Personal taste

## Abstract

Voices elicit rich first impressions of what the person we are hearing might be like. Research stresses that these impressions from voices are shared across different listeners, such that people on average agree which voices sound trustworthy or old and which do not. However, can impressions from voices also be shaped by the ‘ear of the beholder’? We investigated whether - and how - listeners' idiosyncratic, personal preferences contribute to first impressions from voices. In two studies (993 participants, 156 voices), we find evidence for substantial idiosyncratic contributions to voice impressions using a variance portioning approach. Overall, idiosyncratic contributions were as important as shared contributions to impressions from voices for inferred person characteristics (e.g., trustworthiness, friendliness). Shared contributions were only more influential for impressions of more directly apparent person characteristics (e.g., gender, age). Both idiosyncratic and shared contributions were reduced when stimuli were limited in their (perceived) variability, suggesting that natural variation in voices is key to understanding this impression formation. When comparing voice impressions to face impressions, we found that idiosyncratic and shared contributions to impressions similarly across modality when stimulus properties are closely matched - although voice impressions were overall less consistent than face impressions. We thus reconceptualise impressions from voices as being formed not only based on shared but also idiosyncratic contributions. We use this new framing to suggest future directions of research, including understanding idio-syncratic mechanisms, development, and malleability of voice impression formation.

## 1 Introduction

When meeting a person and hearing their voice for the first time, listeners can very quickly form a first impression of what the person they are talking to might be like: Do we think they are an adult; a man; to what extent are they likeable, or attractive? While some of these impressions can be reasonably accurate (e.g., gender), many other impressions do not have a clear link to a ground truth (Foo et al., 2022; Jiang et al., 2024; Todorov et al., 2015). Nonetheless, whether accurate or not, first impressions from voices (and faces) guide and inform our behaviour, such that basic voice and face properties can influence who people vote for in an election (Klofstad, 2016; Mileva et al., 2020; Schild et al., 2022; Tigue et al., 2012), whether a landlord decides to rent a property to a person (Purnell et al., 1999), who we want to affiliate with (Zuckerman and Miyake, 1993), and how harshly criminals are sentenced in court (Chen et al., 2016; Wilson & Rule, 2015).

Research on trait perception from voices usually stresses that impressions are shared across listeners (e.g., Lavan, 2023; Mahrholz et al., 2018; McAleer et al., 2014; Mileva & Lavan, 2023; Rezlescu et al., 2015): That is, listeners are suggested to agree with one another on whether a person sounds, for example, more or less pleasant, friendly, feminine, or older. This focus on the shared nature of impressions has in turn driven theory development, such that theories usually emphasise that the apparent agreement between perceivers could be evidence speaking to listener judgements being based on either common (social) stereotypes (e.g., Aronovitch, 1976; Schirmer et al., 2020; Zuckerman & Driver, 2014) or posit that perception and production have been shaped by evolutionary processes (e.g., Hughes & Rhodes, 2010; Pisanski & Bryant, 2019; Puts et al., 2006). Theoretical work and discussions of the empirical data therefore are almost solely focused on explaining the shared nature of impressions. The focus on shared impressions has not only affected academic research but is also reflected in important public discussion - for example, the media will suggest to women leaders to lower their voice pitch to sound more assertive, a strategy famously adopted by Margaret Thatcher (Brown, 2019). Implicit in this discourse is the idea that it is possible to appeal to ‘most’ listeners, without consideration for individual differences in impressions.

It is, however, very unlikely that listeners always agree with each other for all types of impressions. For example, while many people will agree that, for example, Alan Rickman's voice is pleasant to listen to (cf. Zacharek, 2016), individual listeners may also disagree with this edict. More generally, some listeners, may, for example, have an idiosyncratic but systematic preference for a soft and husky voice while another person prefers a deep and clear voice. This idea of people having idio-syncratic preferences, at least in the visual domain, is well-captured in the folk saying, “beauty is in the eye of the beholder”. Surprisingly, despite being an intuitive concept, the role of idiosyncratic preferences of individual listeners is, however, not a prominent part of theoretical or empirical voice research to date.

This omission of individual listeners is all the more surprising because existing literature *does* routinely examine how the evaluation of individual voices differ from one another from the perspective of voice *production* (e.g., Borkowska & Pawlowski, 2011; Puts et al., 2006; Belin et al., 2017; Schild et al., 2022; Babel, McGuire, & King, 2014; Pisanski & Rendall, 2011). These studies have been very fruitful, asking, for example, why and how different individual voices sound more or less trustworthy, attractive, or dominant to listeners, often linking differences in perceived characteristics to acoustic features (such as average pitch or pitch contour: Ponsot et al., 2018; McAleer et al., 2014). Yet, systematic research examining individual differences in listeners' perceived impressions do not exist to our knowledge. Instead, studies tend to report high inter-rater agreement to confirm that mean ratings derived across participants' individual ratings are capturing valid information (as opposed to e.g., random responses). As a strategy to discover vocal cues that inform perception, this method is ideal. However, by retaining data at the level of the individual voice recordings but not the individual listeners, the resulting conclusions describe individual differences in voice trait production at the expense of understanding individual differences in voice impression formation.

We note some exceptions: In the language attitudes literature, studies look at which social evaluations are associated with different native and non-native accents. Listeners from different language or accent groups (e.g., native vs non-native speakers) show differences in how they evaluate other people based on their accents (Bayard et al., 2001; Hendriks et al., 2023; Boduch-Grabka & Lev-Ari, 2021; see Sharma et al., 2022 for a recent review). Similarly, Tompkinson, Mileva, Watt, & Mike Burton (2024) report that lay listeners do not agree on their assessment of perceived threat conveyed in a voice, thus finding surprisingly limited evidence for shared impressions. The authors then subsample their data to show that small groups of listeners vary in their assessment of threat potential from voices. These studies thus show that impressions of voices can depend on the characteristics of listeners, thus moving some way away from assuming universally shared impressions. However, while intriguing, these studies still focus on group-level differences (e.g., native vs non-native listeners) rather than systematically assessing individual or idiosyncratic differences per se.

Importantly, outside of the voice perception literature, compelling evidence now exists that idiosyncrasy is an important part of impression formation. Using various variance partitioning approaches, studies show that first impressions from faces for example can be characterised by both shared versus idiosyncratic contributions (Albright et al., 1988; Bronstad & Russell, 2007; Germine et al., 2015; Hehman et al., 2017; Hönekopp, 2006; Leder et al., 2016; Martinez et al., 2020; Sutherland, Burton, et al., 2020). Furthermore, studies show that different person characteristics seem to be associated with different profiles of shared versus idiosyncratic contributions to impressions. For example, Hehman et al. (2017) report that “physical” (i.e. readily apparent) characteristics, such as gender typicality and youthfulness, tend to be less idiosyncratic than “trait” or “social” (i.e. inferred) characteristics, such as creativity and competence (see also Albright et al., 1988). Although plausible, it is yet untested if and how these findings translate to voices.

### 1.1 The current study

In the current study, we systematically characterise the idiosyncrasy of person perception from voices across two experiments with just under one thousand individual participants. By quantifying the degree to which voice impressions are idiosyncratic, we address a key gap in the field: current theories of impressions entirely focus on how impressions are shared across listeners, which is likely incomplete (and, arguably, one-sided). We additionally compared voice and face impressions to test for differences and similarities in impression formation across the two modalities, thus tackling the further key theoretical question of how similar or different voice and face perception may be (cf. Belin et al., 2011; Young et al., 2020; Yovel & Belin, 2013) from a novel perspective.

### 1.2 Analytic approach

To address our research questions, we use methods adapted from social psychology and the face perception literatures (e.g., Hehman et al., 2017; Hönekopp, 2006; Kenny, 1994; Martinez et al., 2020; Sutherland, Burton, et al., 2020) by applying a variance partitioning approach to quantify shared (i.e., aspects that different listeners generally agree on) and idiosyncratic (i.e., aspects of that are specific to a listener) contributions to impressions.

Specifically, shared contributions to impressions are captured through the target intra-class correlation coefficient (ICC), which measures how voices vary, (for example) such that some voices sound more attractive than others; while idiosyncratic contributions to impressions are captured through the target*participant interaction ICC, which measures how people vary, (for example) such that one person may find different voices to be attractive than another person (see Hehman et al., 2017; Hönekopp, 2006; Sutherland, Burton, et al., 2020). Our models also include participant ICCs, which are ambiguous: they may measure how participants differ in (for example) showing generally high or low attractiveness judgements, but they could also reflect differences in response behaviours, such as scale use (Hehman et al., 2017; Hönekopp, 2006; Kenny, 1994). Given this ambiguity, we therefore conservatively inferred idiosyncratic contributions only from the interaction ICC in the current study. Finally, our models include a residual error term, which indexes the degree of variance that is not systematic, i.e., any remaining inconsistency in ratings. These four ICC components sum to 1, such that individual components are partially dependent on each other.

## 2 Experiment 1

Experiment 1 first tested whether and how idiosyncratic and shared contributions shape impression formation from voices, and (in comparison) to impression formation of faces. Participants rated 100 short recordings of voices and images of faces for one of 8 characteristics (gender, age, health, attractiveness, dominance, competence, trustworthiness, likeability). These characteristics are important to voice (and face) perception (Lavan, 2023; McAleer et al., 2014; Oosterhof & Todorov, 2008; Sutherland et al., 2013; Lin et al., 2021). We selected characteristics that ranged from directly *apparent* or transparently judged from stimuli (e.g. gender, age, health) – often with some degree of accuracy – to those which cannot be accurately judged from the sound of a voice or the look of a face but are nonetheless readily *inferred* (e.g. trustworthiness, likeability, competence). Note that we do not mean to suggest that apparent and inferred characteristics differ from each other in a categorical, binary manner; instead, all characteristics are probably (to varying degrees) apparent and inferred.

Overall, we predicted that some of the variance in voice impressions is shared across participants and some of the variance will be idiosyncratic to participants. Additionally, we expected that different types of impressions will be more or less shared or idiosyncratic. Specifically, we predicted that characteristics which are apparent from voices (gender, age, health) are mostly shaped by shared rather than idiosyncratic contributions to impressions. Conversely, impressions which are inferred (trustworthiness, friendliness, competence) can be explained by idiosyncratic contributions at least as much as by shared contributions to impressions (based on Albright et al., 1988; Hehman et al., 2017). Given this prediction, attractiveness and dominance impressions from voices, which can be seen as being to similar degrees apparent and inferred in their nature, may show an intermediate profile, being equally driven by shared and idiosyncratic contributions. Thus, described in broader terms across the different characteristics, we expected to see a negative relationship between the amount of variance explained by shared contributions and the amount of variance explained by idiosyncratic contributions of impressions for voices, following Hehman et al. (2017)’s results for faces.

We avoided directional predictions when comparing voice and face impressions given the novelty of this comparison and lack of evidence in the existing literature. We, however, thought it likely that voices and faces would show both similarities (reflecting general processes of impression formation) and differences (reflecting modality-specific processes). Our study hypotheses and methods were pre-registered on the OSF (doi:10.17605/OSF.IO/9DBEP).

### 2.1 Methods

#### 2.1.1 Participants

Our final sample consisted of 498 participants. 245 participants completed the voice task (mean age = 32.3 years, SD = 9.9 years, 102 female, 2 did not provide gender information) and 243 participants (mean age = 32.9 years, SD = 9.4 years, 120 female, 1 did not provide gender information) completed the face task. Thus, 30–32 participants provided ratings for each characteristic. The sample sizes were modelled on previous studies of trait perception (e.g., Hehman et al., 2017; Hönekopp, 2006; Lavan, 2023; Mileva & Lavan, 2023; McAleer et al., 2014). All participants were recruited via Prolific.co and were native speakers of German (matching the voice stimuli used in this expeiment, see *Materials*), aged 18–65, had no self-reported hearing impairments (for voices only), and had normal or corrected-to-normal vision (for faces only).

We excluded 96 additional participants based on our pre-registered criteria: 3 participants failed more than 20% of the in-task attention checks, 2 participants provided more than 80% of the same response per rating scale, and 76 participants (30 for voices, 36 for faces) were unable to identify which characteristic they had been rating immediately after finishing the task (see *Procedure*). For faces, we further excluded 15 participants who accurately recognised any of the faces by name (established at debrief, see procedure). This last criterion was only implemented for faces as they included faces of minor celebrities, while the voice stimuli did not include recordings from any famous people.

#### 2.1.2 Materials

##### 2.1.2.1 Voices

We sampled the first two words from voice recordings of the phrase “Good morning, how are you?” (“Guten Morgen, wie geht es ihnen?”) from 84 voices (42 female, aged 20–60 years at the time of the recording) from the Saarbrücken voice database (Pützer & Barry, n. d.). We chose these types of stimuli as they are well-used in the voice perception literature (e.g., studio-quality recordings of semi-scripted speech). Voices were selected to be spread evenly across the sampled age range, while also being closely matched in age across gender (mean age female: 37.7 yrs., SD = 12.0 yrs.; mean age male = 38.2 yrs., SD = 11.8 yrs). Half of the voices were male and the other half were female. Otherwise, the voices were randomly selected from within the larger database. All selected speakers used an accent that approximates standard German although some regional variation was detectable. All speakers were emotionally neutral. We root-mean-square normalised the recordings across speakers for intensity and converted them into MP3 format.

##### 2.1.2.2 Faces

84 face stimuli (42 female) were sampled from the rated ~2200 faces from the US 10 K ambient image faces database which represents profile pictures taken from the internet, cropped around the face (Bainbridge et al., 2013). We chose this database as it is well-used in the face perception literature. These stimuli were filtered by ratings taken from the original database, for perceived ethnicity (“white”) and emotional content (“neutral”), with half perceived as female (half male). These criteria were used to broadly match the emotional content and regional/ethnic diversity present in the voice stimuli. Faces were otherwise randomly selected. Sampled age ranges were similar across men and women, with the pre-existing ratings of perceived age for these faces also ranging from 20s to 60s, comparable to the voices.

#### 2.1.3 Procedure

The experiment was implemented in Gorilla (Anwyl-Irvine et al., 2020). Participants first read an information sheet and gave informed consent. For voice ratings, participants were asked to wear headphones and to complete the task in a quiet environment. Listeners then completed a basic sound playback check. For face ratings, participants were asked to wear their glasses (if necessary) and to sit at arm's length from their computer screen. Participants calibrated their screen, such that images were presented to them at the same size, independently of screen resolution.

Participants were randomly assigned to complete 1 of the 8 rating tasks (gender, age, health, trustworthiness, dominance, attractiveness, friendliness, or competence) for one modality (faces or voices). Participants used a rating scale from 1 to 9 (e.g., “How attractive is this person?” 1 = “not at all attractive”, 9 = “very attractive”. For gender, 1 ="very feminine" and 9 = "very masculine"; for age 1 = “sounds/looks like a young adult” and 9= “sounds/looks like an old adult”). All stimuli were presented and rated twice by each participant to be able to calculate the ICCs (specifically the interaction ICC; see *Data Analysis*). The rating scale was only shown to participants on their screens after the recording finished playing, such that they listened to the full recording before providing a rating. Face images were presented for the mean duration of the voice recordings (820 ms), also followed by a rating scale being displayed on the screen. Between trials, a fixation cross was shown for 200 ms. The responses were self-timed and there were six attention checks/vigilance trials to help ensure data quality. For these attention checks during the voice ratings task, participants heard a recorded instruction (e.g., “Please select number 1” - in German). For faces, the same instructions were shown as written text. In total, there were 174 trials per participant (84 stimuli * 2 presentations of each face/voice+6 vigilance trials).

Trial order was randomised, such that all stimuli and half of the attention checks were first presented once in fully randomised order. This process was then repeated for the second presentation of all stimuli and the remaining vigilance trials. This procedure prevented the repetitions of the same stimuli from occurring in close succession to one another.

After the rating task, participants completed a debrief questionnaire, where they were asked to identify which characteristic they had just rated, out of all possible traits included in the study. They were also asked to report on any technical issues, whether they paid sufficient attention throughout the task, and were given space to note anything relevant. The experiment took between 5 and 10 min.

#### 2.1.4 Data analysis

To quantify shared versus idiosyncratic contributions to voice (and face) impressions, we calculated ICC(2,1) per rating scale and modality (Shrout & Fleiss, 1979). We achieved this by fitting an intercept-only linear mixed model using *lme4* (Bates, Mächler, Bolker, & Walker, 2014) in R with random effects for the target, participant, and their interaction and calculating how much variance each of these effects explains (see Sutherland, Rhodes, et al., 2020). The ICC(2,1) characterises the variance at the level of the individual participant, the target or stimulus and their interaction (i.e. via single measures as opposed to taking an average). It also measures absolute agreement. We examined shared contributions to impressions by calculating the amount of variance associated with the random effect of the target, i.e., a voice or a face stimulus (target ICC). To examine idiosyncratic contributions to impressions, we calculated the interaction of random effects for target and participants (interaction ICC). We also examined the amount of variance associated with the random effect of the participant (participant ICC), although this type of variance is more difficult to interpret, as noted previously. The remaining variance is the residual error, that is, variance that cannot be attributed to any of the other components and reflects the degree of inconsistency in ratings. ICC values are calculated across participants and stimuli, such that it is not possible to run traditional statistical tests on our data: Only one value is available for the target, participant, interaction ICC, and the residual error, respectively. We, therefore, computed 95% confidence intervals around the different ICCs using the bootMer function from the *lme4* package (Bates et al., 2015) in R (see Sutherland, Rhodes, et al., 2020) to facilitate the interpretation of our results.

We describe broad trends in the data in terms of where different characteristics and/or types of ICCs differ from one another via pairwise comparisons. Where CIs do not overlap – across characteristics and/or across types of ICC – we inferred differences. While there is no clear one-to-one mapping from CIs to e.g. *p*-values, CIs that touch but do not overlap have been shown to be comparable to an α level of *p* =.01 (Cumming, 2013; Huey Tan & Beng Tan, 2010). If desired, this ‘rule of thumb’ may be used to roughly align our findings with p-value based inferences. For reference, all ICC values and their 95% CIs are reproduced in Supplementary Table 1.

### 2.2 Results and discussion

#### 2.2.1 Voices

**Fig. 1:**
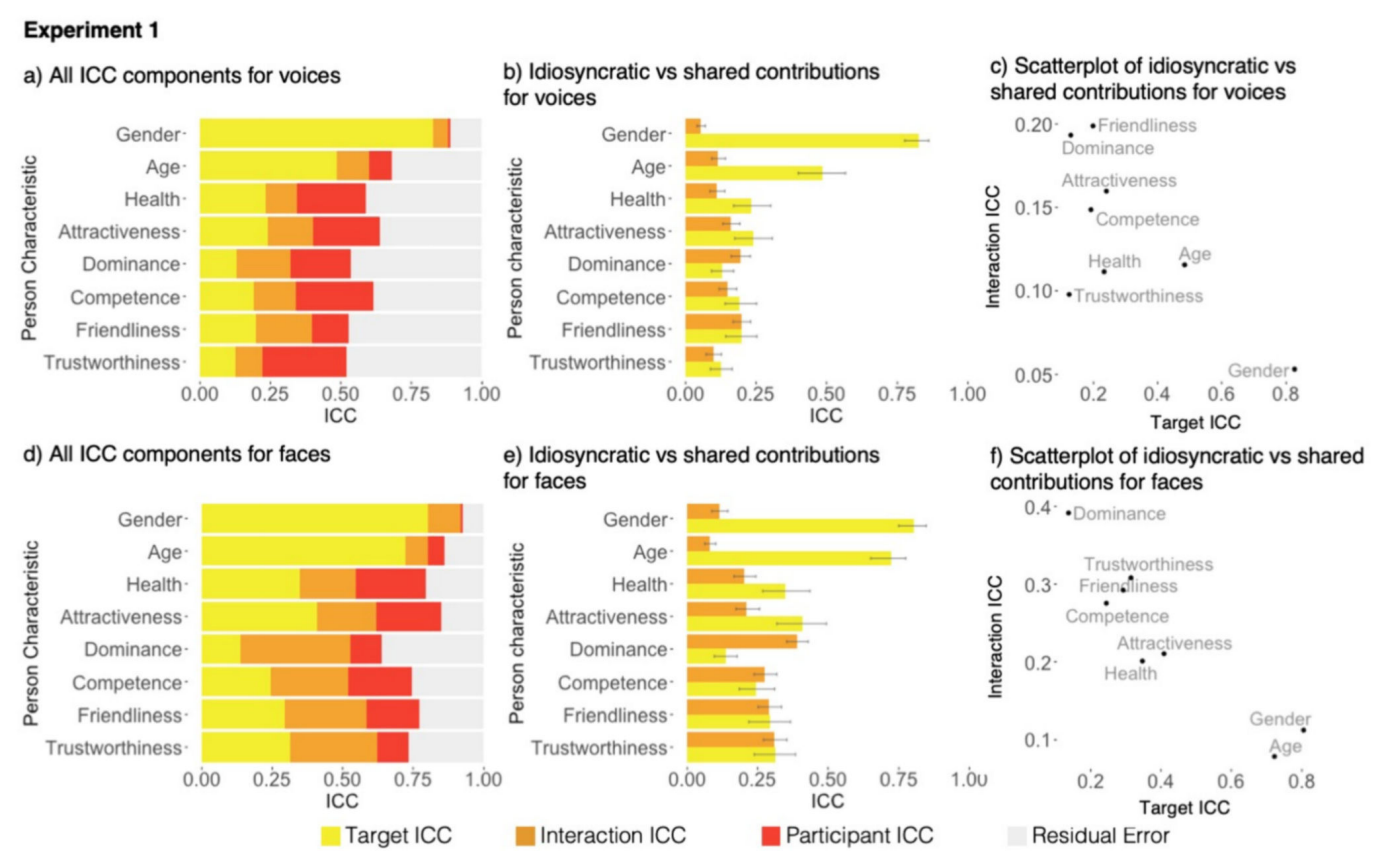
Illustration of the data from Experiment 1. a) Stacked bar chart illustrating the variance accounted for impressions of voices as measured by different Intraclass Correlation Coefficients (ICCs): Target ICC (indexing shared contributions to impressions), Interaction ICC (indexing idiosyncratic contributions to impressions, Participant ICC, Residual error. b) Bar chart comparing the variance accounted for by target ICCs and interaction ICCs for voices, error bars show 95% confidence intervals. c) Scatterplot illustrating the relationship between interaction ICCs and target ICCs for voices. d) Stacked bar chart illustrating the ICC components for faces. e) Bar chart comparing the variance accounted for by target ICCs and interaction ICCs for faces, error bars show 95% confidence intervals. f) Scatterplot illustrating the relationship between interaction ICCs and target ICCs for faces.

As predicted, all voice impressions are characterised by both idio-syncratic and shared contributions (see Fig. 1). How idiosyncratic these voice impressions are, differs for each characteristic. To quantify which aspects of impression formation contribute most to the different characteristics, we compared shared (target ICC) vs idiosyncratic (interaction ICC) contributions to impressions. We found that for impressions of characteristics primarily that are directly apparent (gender, age and health), shared contributions exceed idiosyncratic contributions as indicated by the lack of overlap between 95% confidence intervals of the target and interaction ICCs (Fig. 1a and b). For the remaining impressions (attractiveness, dominance, competence, trustworthiness, and friendliness), shared and idiosyncratic contributions appeared similar, with CIs for the target and interaction ICCs overlapping. This pattern therefore confirmed our prediction that impressions of person characteristics which are directly apparent from physical cues are shaped to a larger extent by shared contributions. Impressions of inferred characteristics are shaped to similar degrees by idiosyncratic and shared contributions.

We observed a negative relationship between target and interaction ICCs (Fig. 1c; see also Hehman et al., 2017), although this relationship is mainly driven by gender forming an outlier. Overall, however, when only looking at the absolute contribution of shared aspects of impression formation, apparent person characteristics tend to show larger shared contributions than inferred person characteristics, as predicted. The opposite picture emerged when looking at idiosyncratic contributions to impressions: We observed that idiosyncratic contributions tended to be larger for impressions of inferred person characteristics compared to apparent person characteristics. As predicted, attractiveness impressions indeed showed an intermediate profile, where shared contributions narrowly exceeded idiosyncratic contributions, although CIs overlap. Against predictions, dominance, however, behaved like an inferred characteristic (instead of showing an intermediate profile), with idio-syncratic contributions to impressions being larger than the shared contributions to impressions. This finding for dominance was somewhat surprising, given that dominance perception in the voice literature is often seen as being closely linked to the perception of physical or apparent properties, such as formidability and strength (Armstrong et al., 2019; Aung & Puts, 2020; Puts et al., 2006).

#### 2.2.2 Faces

The data for face impressions are visualised in Fig. 1d-f. In line with our findings for voices, and replicating previous research (Albright et al., 1988; Hehman et al., 2017), both shared and idiosyncratic contributions also characterise impressions formed from faces. For the three apparent characteristics, gender, age and health, as well as attractiveness, impressions were mostly shaped by shared contributions as indicated by the lack of overlap between 95% confidence intervals of the target and interaction ICCs (Fig. 1d and e). For competence, trustworthiness, and friendliness, impressions were shaped by shared and idiosyncratic contributions to a similar degree, with CIs for the target and interaction ICCs overlapping. For dominance impressions, idiosyncratic contributions were larger than the shared contributions. Thus, impressions of apparent person characteristics for faces are also shaped to a larger extent by target characteristics, while impresssions of more inferred person characteristics were shaped by shared and idiosyncratic aspects to similar degrees.

As for voice impressions, we also found a negative relationship between shared and idiosyncratic contributions to face impressions (Fig. 1f). Specifically, there were again more shared contributions for apparent person characteristics compared to inferred characteristics, while the opposite is true for inferred person characteristics, where idiosyncratic contributions were larger. As for voices, this negative relationship is mainly driven by gender and also age forming outliers with having much larger shared contributions than the remaining characteristics.

#### 2.2.3 Comparing faces and voices

**Fig. 2:**
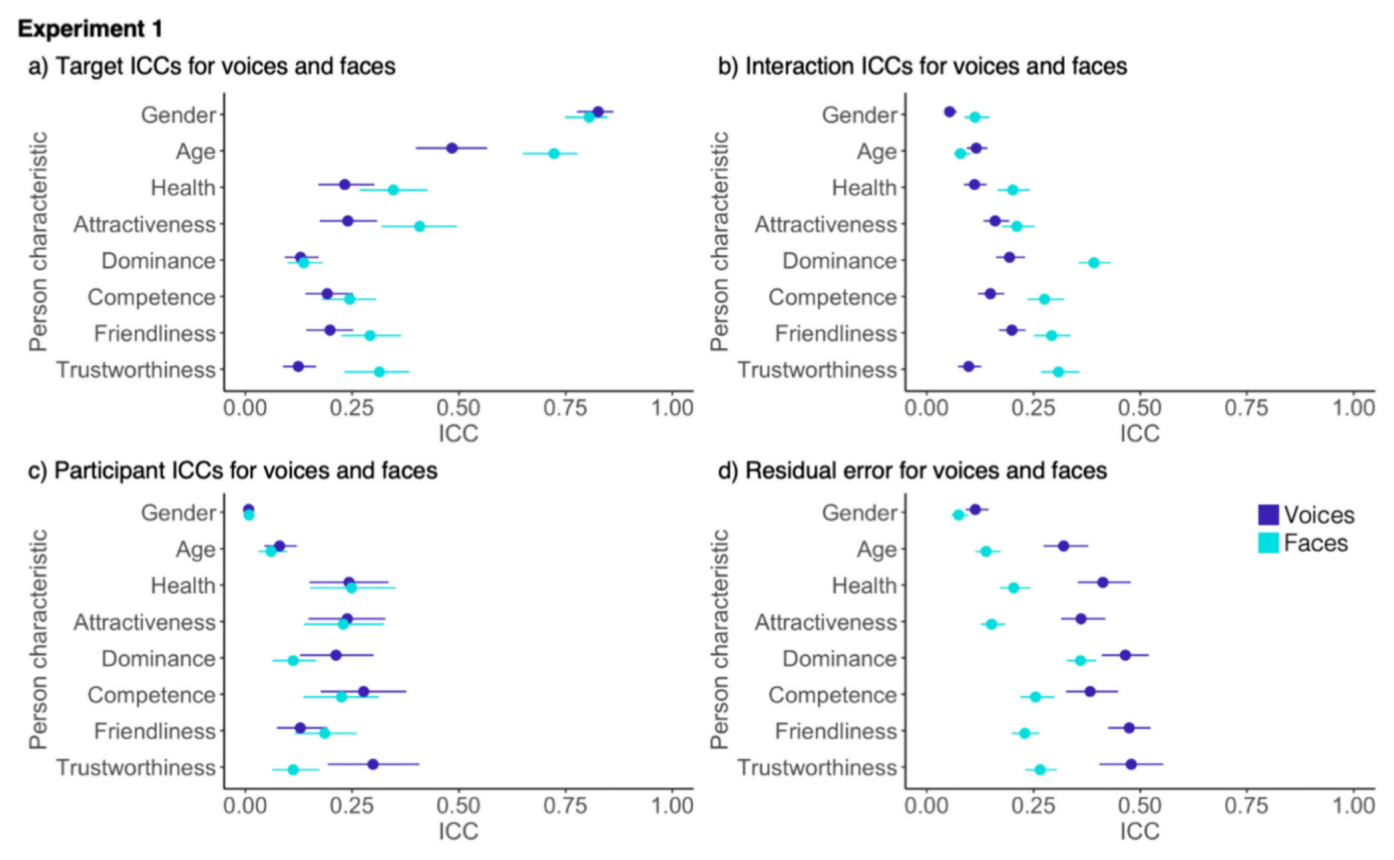
Comparison of ICC components for voices and faces from Experiment 1 with a) showing the target ICCs (shared contributions to impressions), b) showing the Interaction ICCs (idiosyncratic contributions to impressions, c) showing the participant ICCs and d) showing the residual error. Error bars show 95% confidence intervals around the mean.

Shared contributions to impressions were comparable for voices and faces except for age, attractiveness, and trustworthiness, where target ICCs for faces exceeded those of voices (Fig. 2a). For idiosyncratic contributions to impressions, however, interaction ICCs were consistently larger for faces than for voices for all characteristics apart from for attractiveness, where confidence intervals overlapped (Fig. 2b). This pattern suggests an overall more pronounced role of personal taste for faces compared to voices in this experiment.

There were also differences between voice and face impressions for participant ICCs and the residual error. For participant ICCs, ICCs were similar for faces and voices with overlapping confidence intervals – except for trustworthiness, where contributions of participant characteristics were bigger for voices than for faces (Fig. 2c). The residual error was bigger for voices for all characteristics apart from gender, where contributions were similar for faces and voices (Fig. 2d). These results suggest that, although participant ICCs explain a similar amount of variance for face and voice impressions, impressions formed based on voices were generally less consistent.

Experiment 1 for the first time showed that both shared and idiosyncratic contributions shared impressions of person characteristics from voices. Increased shared contributions (compared to idiosyncratic contributions) were found for more apparent person characteristics relative to more inferred person characteristics. Set against an overall pattern of broad similarities between voice and face perception, there were some notable differences between modalities. Specifically, we found that the idiosyncratic contributions to impressions were generally smaller for voices compared to faces. Similarly, shared contributions to impressions were smaller for voices than for faces for age, trustworthiness, and attractiveness. Finally, the residual error in models was consistently larger for voices compared to faces, which may speak to voice impressions being overall less consistent (across targets, perceivers, or both).

## 3 Experiment 2

The stimuli used in Experiment 1 are reflective of stimuli frequently used in voice and face research (e.g., clean, semi-spontaneous recordings of greetings; static images of faces). With these stimuli, we were therefore able to quantify how shared and idiosyncratic contribute to impressions from voices and faces, respectively, in line with the types of stimuli used in previous experiments on impression formation. However, Experiment 1 also necessarily included two different sets of identities and two sets of stimuli that differ in their overall properties (static vs dynamic), which could have affected the observed patterns of shared and idiosyncratic contributions to impressions.

In Experiment 2, we thus aimed to replicate Experiment 1, however, now using a stimulus set that closely matches the properties of the face and voice stimuli across modalities. Instead of using static, variable face images and dynamic, highly-controlled voice recordings, from two separate sets of identities, we used naturally-varying dynamic voice and face stimuli that were created from within the *same* audio-visual video recording in Experiment 2. The voice and face stimuli thus featured the same identities across modalities and were also well-matched across any number of other incidental features present in the stimuli.

In Experiment 2, as in Experiment 1, we had two main predictions. First, for voices and faces alike, we predicted that impressions have both shared and idiosyncratic contributions. Second, we predicted that different types of impressions for voices will show different profiles of shared versus idiosyncratic contributions, such that impressions of apparent person characteristics (gender, age, health) would be more driven by shared contributions than idiosyncratic taste, while impressions of inferred person characteristics (trustworthiness, friendliness) should be relatively equally shaped by shared and idiosyncratic contributions. We had predicted in Experiment 1 that attractiveness and dominance impressions from voices would show a profile of contributions of shared versus personal taste that falls between the profiles seen for apparent and inferred characteristics. We kept our prediction for attractiveness; however, dominance behaved more like a more inferred characteristic. Experiment 2 tested whether this unpredicted result generalised to a new stimulus set and participant sample.

Through using these new stimuli, we could also examine which properties of the stimuli might affect shared versus idiosyncratic contributions (and inconsistency) by comparing Experiments 1 and 2. For example, the stimuli in Experiment 2 included more variability for voices compared to the stimuli in Experiment 1, while the face stimuli were now dynamic (but similarly variable compared to the static face stimuli in Experiment 1). Thus, if the degree of overall variability in the stimuli increases shared and/or idiosyncratic contributions, we expected to see those target and/or interaction ICCs increase for voices in Experiment 2 due to the increased variability. If conversely, the dynamic nature of the stimuli matters, we expected the interaction ICC and/or the residual error for faces to increase in Experiment 2. The interaction ICC could increase for faces because participants could now base their impressions on different dynamic cues: For example, in a video that starts with a person smiling, followed by a more neutral expression, one participant might form an impression based on an initial smile, while another participant might prioritise the later and more neutral expression. Alternatively, the residual error would increase if (for example) a participant is more influenced by the friendly smile when first rating the video clip but then is more influenced by the neutral face for the second rating. Our second study was also pre-registered (https://osf.io/nfsgx/?view_only=68e930afde04475d81cc16a22667456e).

### 3.1 Methods

#### 3.1.1 Participants

495 participants were included in the final sample. 245 participants (mean age = 39.2 years, SD = 12.2 years, 110 were female, 1 did not provide gender information) completed the voice rating task and 240 participants (mean age = 49.3 years, SD = 12.4 years, 137 were female) completed the face rating task. Thus, 29–32 participants provided ratings for each person characteristic. All participants were again recruited via Prolific.co. The stimuli in Experiment 2 sampled speech in English and we therefore recruited English-speaking participants born and currently resident in the UK, as opposed to German speakers for Experiment 1. Since stimuli were sampled from celebrities that were primarily known in Canada and Australia, the regional restriction to the UK decreased the likelihood of participants recognising these celebrities (see also exclusion criteria). 87 further participants were excluded from the original sample tested (*N* =582): Of these, 3 failed more than 20% of the in-task attention checks, 1 participant provided more than 80% of the same response per rating scale, and 61 participants (30 for voices, 31 for faces) were unable to identify the characteristic they had rated at debrief. For faces, we additionally excluded 22 participants who accurately recognised any of the faces by name (also established via debrief). None of the voices were recognised by name.

#### 3.1.2 Materials

We used a stimulus set of 72 voice and face stimuli derived from the same short audiovisual recording of a ‘local celebrity’ from Canada or Australia, such as TV presenters or athletes. This stimulus set was developed for another study (Smith et al., 2023). In these audiovisual clips, the celebrities were shown in a broadly frontal pose talking to the camera as themselves as in an interview (i.e., not acting or reading from a script). Actors appear to be aged between 20 and 40 years. All clips were taken from videos uploaded to YouTube. Each clip lasted between 2 and 3 s, during which the celebrity produced a short meaningful utterance, for example, “I was about seventeen”. Spoken utterances thus differed across stimuli. Background noise was minimal, and no other voices were audible. The mean duration of the stimuli was 2.58 s (SD = 0.51 s). No other voices or background music are audible.

To create the face stimuli used in Experiment 2, videos were cropped using an online video cutter (https://online-video-cutter.com/) to show the head and shoulders (3:4 aspect ratio) to a height of 300 pixels. The audio track was muted, such that the voice was not audible. To create the voice stimuli, we extracted only the audio track from the audiovisual clip. These audio tracks were then normalised for peak intensity across all clips.

#### 3.1.3 Procedure & data analysis

Experiment 2 was otherwise identical to Experiment 1 and the same experimental design, sample size, exclusion criteria, and the basic statistical analysis strategies were used.

### 3.2 Results

#### 3.2.1 Voices

**Fig. 3:**
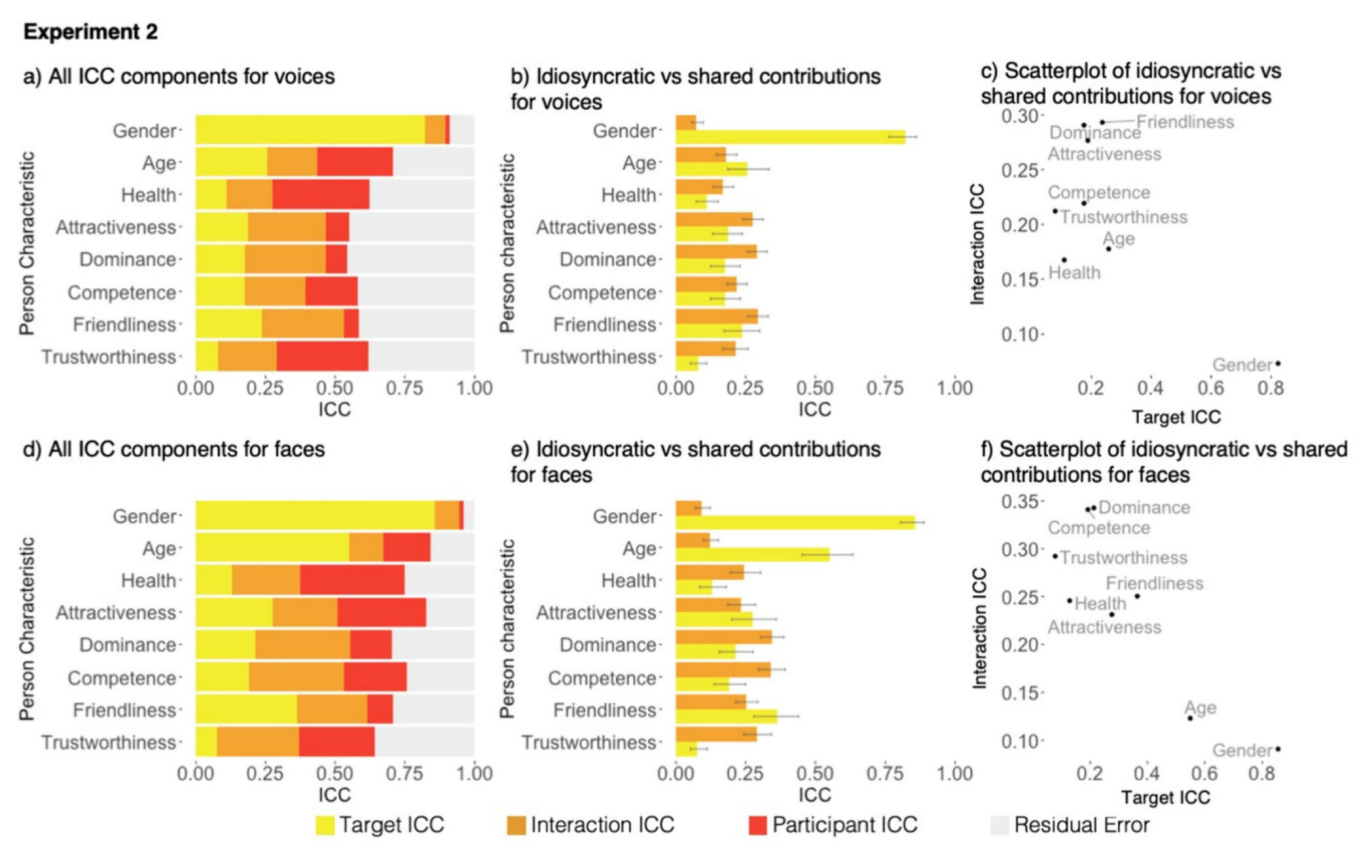
Illustration of the data from Experiment 2. a) Stacked bar chart illustrating the variance accounted for impressions of voices as measured by different Intraclass Correlation Coefficients (ICCs): Target ICC (indexing shared contributions to impressions), Interaction ICC (indexing idiosyncratic contributions to impressions, Participant ICC, Residual error. b) Bar chart comparing the variance accounted for by target ICCs and interaction ICCs for voices, error bars show 95% confidence intervals. c) Scatterplot illustrating the relationship between interaction ICCs and target ICCs for voices. d) Stacked bar chart illustrating the ICC components for faces. e) Bar chart comparing the variance accounted for by target ICCs and interaction ICCs for faces, error bars show 95% confidence intervals. f) Scatterplot illustrating the relationship between interaction ICCs and target ICCs for faces.

Like in Experiment 1, both shared and idiosyncratic contributions shape voice impressions (Fig. 3a) and different patterns emerged again for different person characteristics. For gender impressions (only), shared contributions exceeded idiosyncratic contributions as indicated by the lack of overlap between 95% confidence intervals of the target and interaction ICCs (Fig. 3b). For age, health, competence, and friendliness, shared and idiosyncratic contributions to impressions contributed to similar degrees, with CIs for the target and interaction ICCs overlapping. For attractiveness, dominance, and trustworthiness, idiosyncratic contributions shaped impressions more than shared contributions. Thus, while Experiment 1 suggested that impressions of apparent characteristics are more driven by shared than idiosyncratic contributions to impressions, this pattern did not occur in Experiment 2 for voices. These data also did not show a clear negative relationship between target and interaction ICCs (Fig. 3c). We note that a similar decrease in shared contributions to impressions for age and health was also evident for faces between Experiment 1 and Experiment 2, thus we interpret these differences between experiments as being a result of the stimulus sampling strategy (i.e., sampling a smaller range of age in Experiment 2; see below for details).

**Fig. 4:**
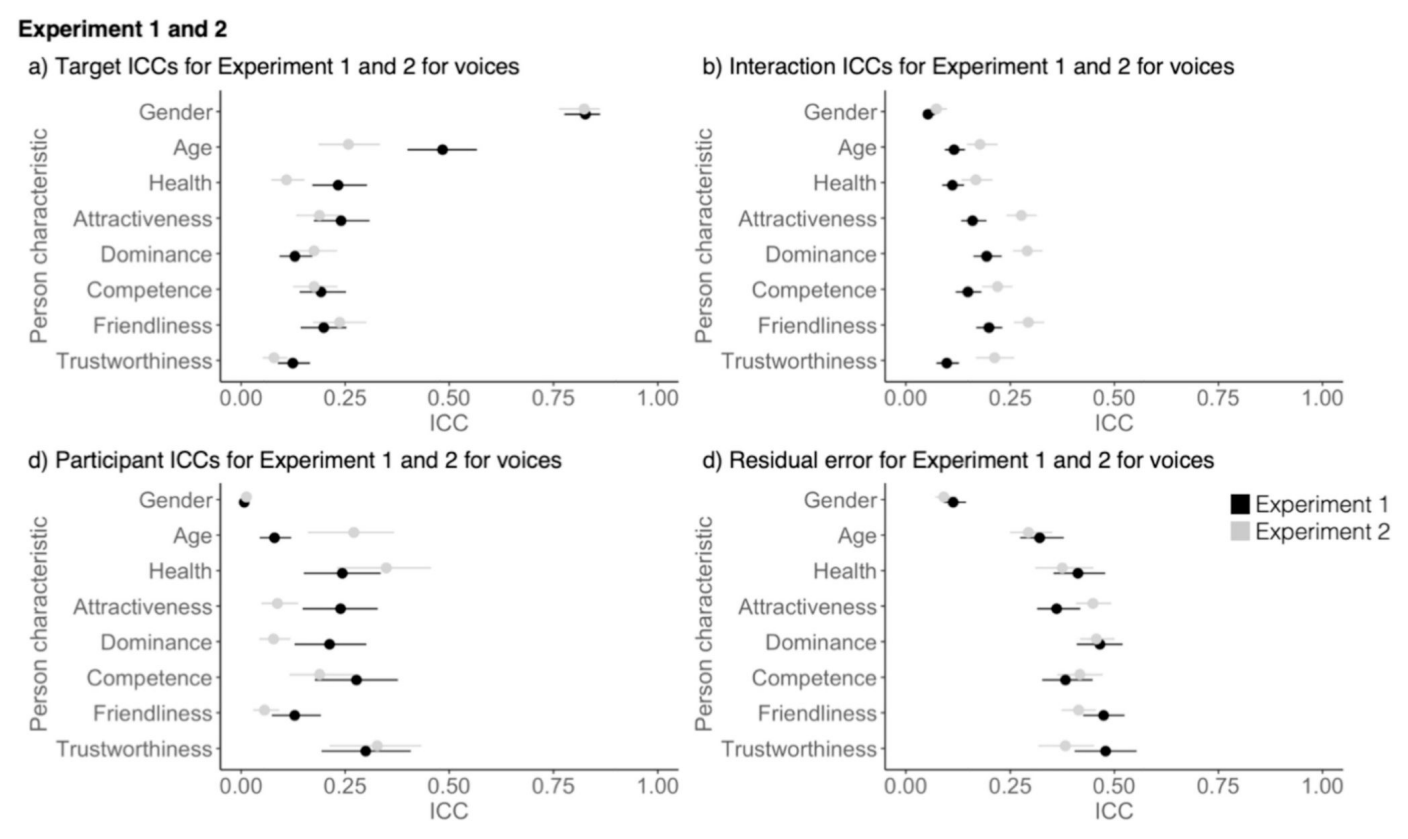
Comparison of ICC components for voices from Experiment 1 and Experiment 2 with a) showing the target ICCs (shared contributions to impressions), b) showing the Interaction ICCs (idiosyncratic contributions to impressions), c) showing the participant ICCs and d) showing the residual error. Error bars show 95% confidence intervals around the mean.

When directly comparing voice impressions for Experiments 1 and 2 (Fig. 4), shared contributions to impressions were higher for two of the apparent characteristics, age, and health, in Experiment 1 compared to Experiment 2. Furthermore, idiosyncratic contributions shaped impressions more in Experiment 2 compared to Experiment 1 for all characteristics. Given the increase in idiosyncratic contributions across all characteristics, it is likely that this increase was brought about by general stimulus properties, most likely through sampling more naturalistic voice recordings. Perhaps, participants gained access to additional information from which they were able to form more consistent idiosyncratic impressions of voices (see Hehman et al., 2017 for a similar finding for faces). Participant ICCs in Experiment 2 were higher for age and lower for attractiveness and dominance. There were no differences in the residual error between Experiments 1 and 2. For all other characteristics, target and participant ICCs and the residual error were similar across Experiment 1 and Experiment 2.

#### 3.2.2 Faces

The data for face impressions are visualised in Fig. 3d-f. Impressions formed from faces were again characterised by both shared and idiosyncratic contributions, replicating Experiment 1 and previous literature.

For gender and age impressions, the shared contributions exceeded idiosyncratic contributions (Fig. 3d and e). For attractiveness and friendliness, shared and idiosyncratic contributions were similar, while for health, dominance, competence, and trustworthiness the shared contributions were larger than idiosyncratic contributions. Therefore, apparent characteristics, particularly age and gender, were shaped to a larger extent by shared contributions, while inferred characteristics were mainly shaped by idiosyncratic contributions. Consequently, we still observed a negative relationship – that was mainly driven by age and gender – between idiosyncratic and shared contributions to impressions formed from faces, as in Experiment 1 (Fig. 3f).

**Fig. 5:**
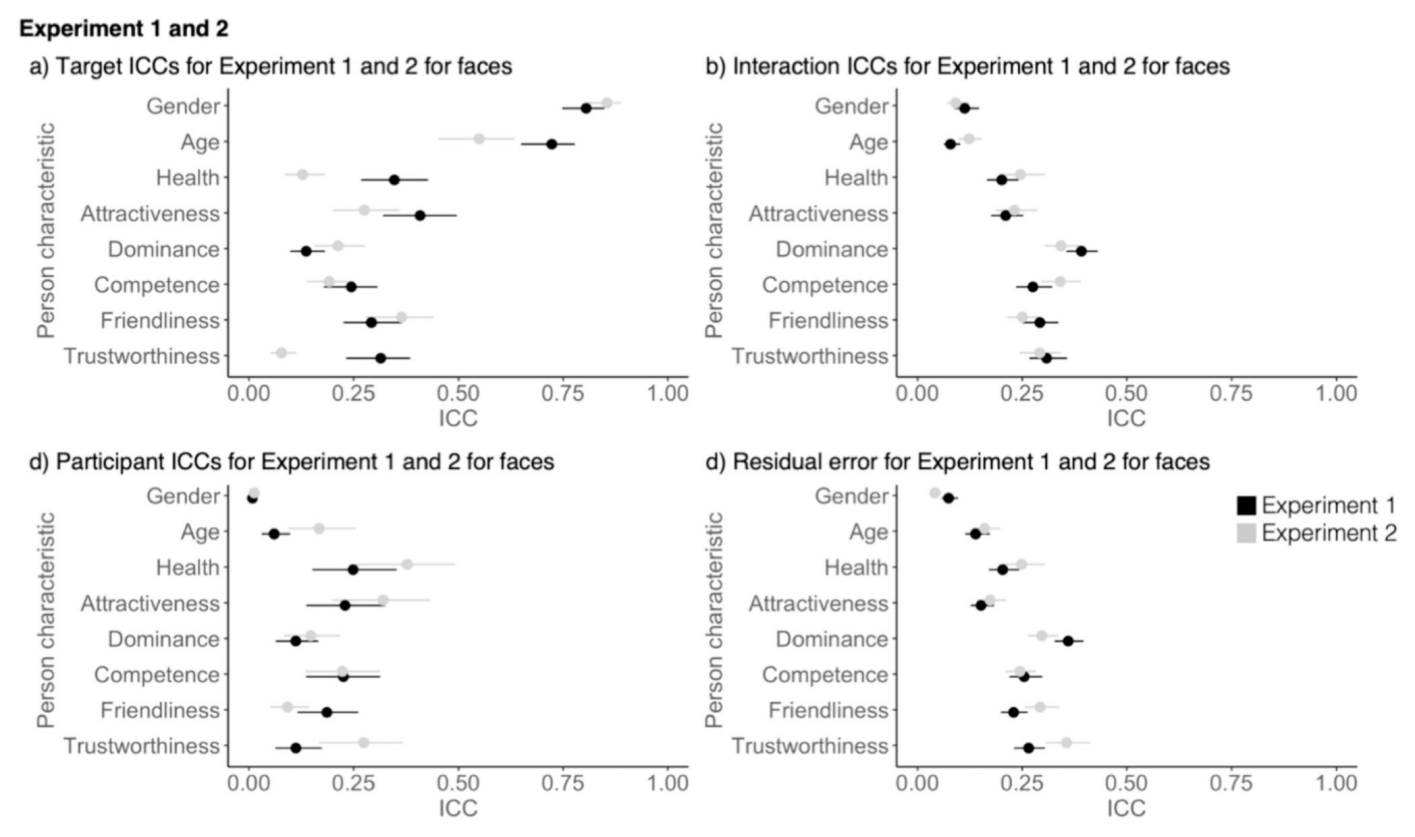
Comparison of ICC components for faces from Experiment 1 and Experiment 2 with a) showing the target ICCs (shared contributions to impressions), b) showing the Interaction ICCs (idiosyncratic contributions to impressions), c) showing the participant ICCs and d) showing the residual error. Error bars show 95% confidence intervals around the mean.

In contrast to impressions from voices, no systematic differences were apparent for faces when comparing the data across Experiments 1 and 2 (see Fig. 5). The idiosyncratic contributions to impressions (i.e. interaction ICCs) were similar across all person characteristics for faces in both experiments. As for voices, shared contributions (i.e., target ICCs) were lower for age, health, and trustworthiness in Experiment 2. Finding that the shared contributions to impressions change in the same way between experiments for both faces and voices further underlines that these changes likely indeed reflect differences in the stimulus sampling strategy (i.e., Experiment 2 covering a smaller age range). Both participant and residual error ICCs were also comparable across Experiments 1 and 2 for all person characteristics. Importantly, we conclude that introducing dynamic faces in Experiment 2 did not systematically affect how impressions of faces are formed.

#### 3.2.3 Comparing faces and voices within Experiment 2

**Fig. 6:**
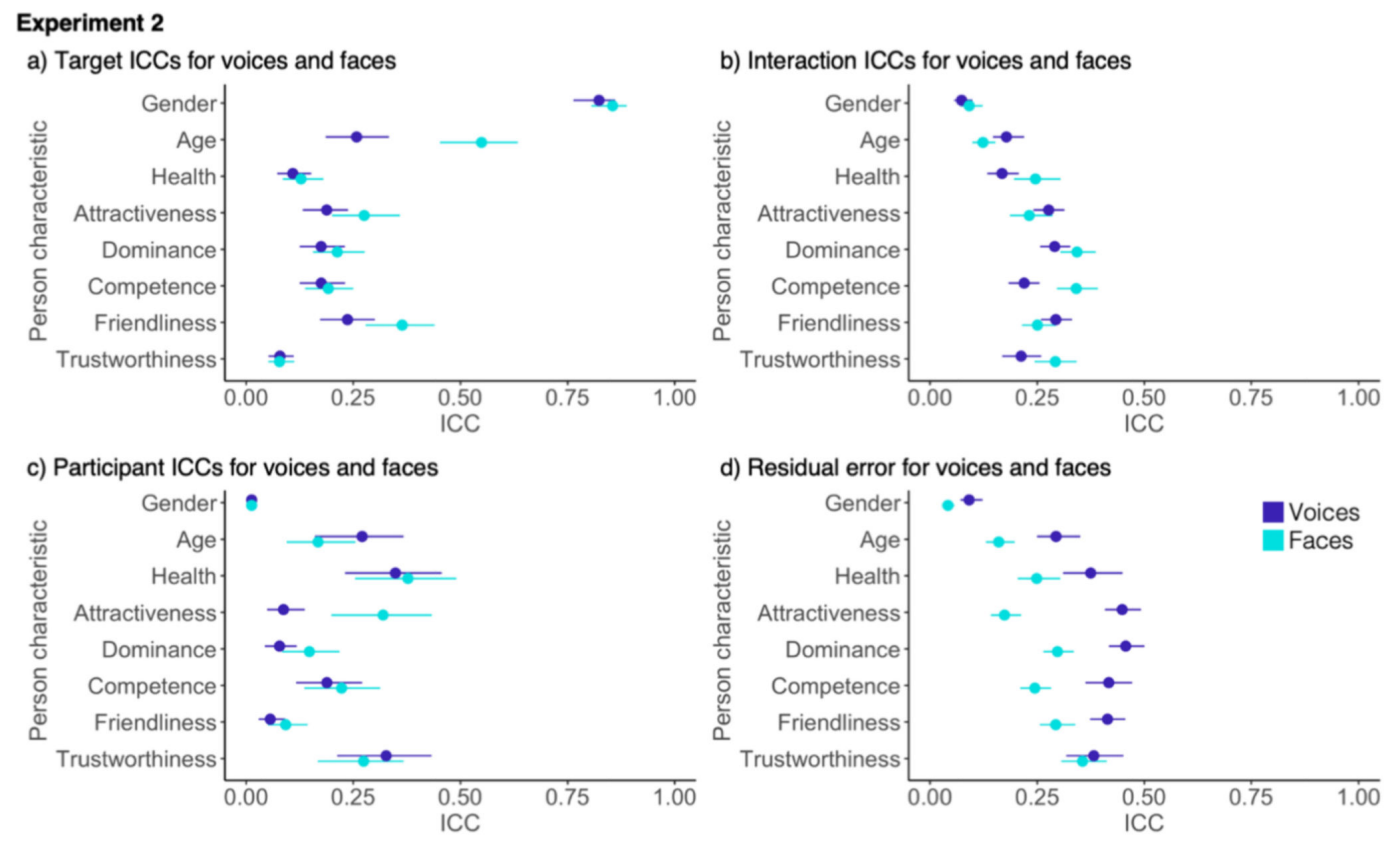
Comparison of ICC components for voices and faces from Experiment 2 with a) showing the target ICCs (shared contributions to impressions), b) showing the Interaction ICCs (idiosyncratic contributions to impressions), c) showing the participant ICCs and d) showing the residual error. Error bars show 95% confidence intervals around the mean.

There were no systematic differences between the target, interaction and participant ICCs for faces and voices in Experiment 2 (Fig. 6). Shared contributions (i.e. target ICCs) were comparable for voices and faces except for age, where target ICCs for faces exceeded those of voices. As in Experiment 1, idiosyncratic contributions (i.e. interaction ICCs) were similar for most person characteristics for faces and voices, with interaction ICCs for faces only exceeding those for voices for competence. Participant ICCs were likewise similar for faces and voices, except for attractiveness, where contributions of participant ICCs were larger for faces than for voices.

However, also as reported in Experiment 1, there was still a systematic overall difference between face and voice impressions: voices showed larger residual errors than faces for all characteristics apart from trustworthiness, where contributions were similar for faces and voices. The overall larger residual error for voices therefore does not seem to be susceptible to changes in stimulus properties as modified across experiments. Given that the residual error indexes the degree of inconsistency of ratings that cannot be explained otherwise, this finding suggests that person-related information is generally less reliably perceived from voices than from faces, independently of stimulus properties. Similar observations have been reported for identity perception, where e.g. identity recognition is in general more error-prone and more prone to disruption for voices than for faces (see Stevenage & Neil, 2014; Young et al., 2020). It is intriguing to observe similar patterns for impression formation, in particular because we make no link or claims to the accuracy of impressions.

Overall, Experiment 2 replicated the core findings from Experiment 1 with much more closely matched and naturalistic stimuli. Again, impressions showed both shared and idiosyncratic contributions, with the weighting of these contributions to impressions depending on the specific person characteristic. The most important additional finding from Experiment 2, however, was that impressions from voices and faces were similarly driven by shared and idiosyncratic contributions when stimuli were well-matched. The only remaining systematic difference in how impressions are formed between modalities was therefore the overall larger residual error for voices.

There were additionally some notable differences between the two experiments, mainly regarding age and health. We speculate that these differences are linked to specific properties of the stimulus sets. For example, impressions of apparent characteristics, specifically age and health, showed less shared contributions (indexed by target ICCs) in Experiment 2 than in Experiment 1. We note that the stimuli in Experiment 2 were sampled from a narrower age range (20s–40s) than in Experiment 1 (20s–60s). This change will have reduced the variability in perceived age in Experiment 2 (a similar change was found for other target properties in Hönekopp, 2006, who examined ethnicity; and Hehman et al., 2017, who tested expression). The same is likely true for the range of perceived health sampled between Experiments 1 and 2, given the stereotyped association of age and health. We suggest that most changes in the degree of shared contributions to impressions can be explained by sampling a new set of identities with different demographic characteristics.

## 4 General discussion

Across two experiments, for the first time, we quantified the shared and idiosyncratic aspects of impression formation from voices, compared these results to impressions from faces, and described how stimulus properties (such as differences in naturalistic variability) affect how much shared or idiosyncratic contributions shape impressions. Critically, we find converging evidence that idiosyncratic contributions play a substantial role in impression formation from voices: the idiosyncratic contributions to impressions were often on par with the shared contributions, especially for inferred person characteristics (trustworthiness, friendliness). Indeed, shared contributions to impressions *only* exceeded idiosyncratic contributions for gender and age, characteristics which are more readily apparent from voice signals themselves.

Comparing across modalities, we found that shared and idiosyncratic contributions drive impressions from voices and faces to similar degrees, with impressions from voices, however, being less internally consistent. These findings tie in well with the wider literature of voice and face perception, which tends to stress that there are many similarities (alongside some differences) in how voice and face perception is achieved (e.g., Belin et al., 2011; Young et al., 2020; Yovel & Belin, 2013). Finding less consistency in person-related impressions can also be seen as converging evidence in line with the finding that voice identity perception is less robust and accurate than face identity perception (e.g., Stevenage et al., 2013; Young et al., 2020). Our findings may suggest that this decreased robustness in voice perception extends beyond identity, to the perception of (m)any person characteristics from voices.

### 4.1 The importance of idiosyncratic contributions to impression formation

Overall, it is perhaps surprising that existing work has not already accounted for these idiosyncratic contributions – and thus effectively individual differences - in listeners. Our findings thus have implications for how first impressions are conceptualised in the existing literature: To date, studies tend to primarily model first impressions as being shared across listeners either explicitly or implicitly (Baus et al., 2019; Belin et al., 2017; Groyecka-Bernard et al., 2022; Lavan, 2023; McAleer et al., 2014; Mileva & Lavan, 2023). While the shared nature of impressions is certainly an important and compelling aspect of impression formation, and interesting research questions revolving around the shared nature of voice impressions are being asked, the literature is currently effectively side-lining another substantial contributor, that of individual differences, to first impressions. Future theoretical and empirical work needs to approach vocal impression research to take account of both shared and idiosyncratic contributions to impressions. While taking such an approach would require changes to experimental paradigms and analyses (e.g., repeated presentation of stimuli), it would open up a number of fruitful lines of inquiry. For example, future research may tackle questions of when and how idiosyncratic contributions emerge in development, and whether or not they are governed by the same influences and mechanisms that govern shared contributions. Some of these questions have already started to be addressed in the face perception literature. Here, twin studies, for example, find that idiosyncratic contributions to impressions of faces can be linked to personal, unshared environmental factors and social learning, rather than being strongly driven by inherited genetic variation (Germine et al., 2015; Sutherland, Burton, et al., 2020). At the same time, however, impressions from faces for an individual perceiver can be mapped onto the same underlying dimensions that are apparent in the group-level data, further highlighting that even when accounting idiosyncratic contributions, we nonetheless perceive faces within a – perhaps learned – shared structure or social reality (Lin et al., 2021; Sutherland, Rhodes, et al., 2020).

We also note that our study likely understates the role of idiosyncratic contributions. We adopted a conservative approach to quantifying idiosyncrasy in impressions, solely basing our estimation of idiosyncratic contributions on a definition that sees these as an interplay of a participant's individual evaluation of a specific voice (e.g., one listener preferring low-pitched voices while another prefers high-pitched voices). This definition of idiosyncrasy is captured in our analysis by the interaction ICC. However, in the face perception literature, some researchers have argued that other aspects of idiosyncratic contributions may also be reflected in the data via other patterns: for example, overall differences in judgement such that one participant may judge all voices as sounding equally unattractive while another person may find all voices attractive (e.g. selecting ratings at the higher end). These aspects of idiosyncratic contributions are also meaningful and would be captured in the participant ICC (e.g., Hönekopp, 2006 for a discussion). However, it is also possible that these patterns simply reflect response bias (i.e. tending to select ratings towards the lower or higher end of the scale), which is not linked to idiosyncratic contributions. This ambiguity limits the interpretability of the participant ICC, such that we have chosen to not include this measure in our definition of idiosyncratic contributions. If we had taken a less conservative interpretation and included participant ICCs, the idiosyncratic contributions to impressions become even more pronounced in our study. In fact, idiosyncratic contributions would reliably exceed the shared contributions for most person characteristics when adopting this more liberal approach.

### 4.2 Key moderators of idiosyncratic and shared contributions to voice and face impressions

Our findings also highlight key factors that affect how much these two types of contributions shape impressions: For voices, more variable stimulus materials (e.g., naturally varying voice recordings) compared to highly controlled stimulus materials increased idiosyncratic contributions. Our results therefore suggest that researchers interested in voice and face perception need to carefully consider how far their results can be generalised in light of their stimulus choices (see Hönekopp, 2006 for a discussion of this point with regard to faces). In many cases where studies intend to draw conclusions about the circumstances most vital to everyday life (such as hearing a voice on the phone, through an online call, or meeting offline), using naturalistic stimuli is the most useful.

We also found that the relative importance of idiosyncratic contributions to impressions depended on which person characteristic are evaluated. For example, we observed that shared contributions to impressions were especially high for characteristics for which the accuracy of perception can be high (e.g., such as gender, and under some circumstances, age; see Owren et al., 2007 and Moyse, 2014). Indeed, accuracy by definition requires consensus among perceivers. For person characteristics where shared contributions accounted for most of the (explainable) variance in impressions (e.g., for gender perception, as also found for faces, Hehman et al., 2017), idiosyncratic contributions were at times negligible. However, when the (perceived) variability in a certain person characteristic was decreased due to stimulus sampling strategies (for example, by sampling a smaller range of ages in Experiment 2), shared contributions also decreased.

While we show compelling evidence for the importance of both idiosyncratic and shared contributions to impressions, these contributions are shaped by additional factors, such as (perceived) stimulus variability and the specific person characteristics evaluated. How and how much idiosyncratic and shared contributions respectively shape impressions is therefore not fixed but likely depends on the perceivers, stimuli, and specific perceptual task.

### 4.3 Future directions

Much future work is needed to redress the balance in the field of impression formation, from solely addressing questions around the shared nature of impressions to also incorporating the concept of idiosyncratic contributions in impression research. In addition to examining the developmental, genetic, and social origins of idiosyncratic contributions (cf. Germine et al., 2015; Siddique et al., 2022; Sutherland, Burton, et al., 2020) and mapping how different factors influence idiosyncratic contributions in first impressions, future research also needs to step beyond *first* impressions to establish how impressions are updated and change over repeated exposures in both shared and idiosyncratic ways. To date, there is only limited experimental work in the voice perception literature exploring when and how first impressions transform into a lasting impression of a familiar person, leaving many core questions unanswered: For how long and to what degree can impressions be changed? To what extent are longer-term changes in impressions dependent on the characteristics of the listener (relatively stable differences in e.g., in- and out-group perception versus more fleeting factors such as mood, fatigue or current goals) compared to differences in the behaviour of the perceived voice? How do listeners combine different types of information that emerged over different time courses (vocal, linguistic, visual, etc.) into a coherent impression?

Finally, when starting to consider that both shared and idiosyncratic contributions can shape first impressions, work on impression formation needs to consider clear definitions of what constitutes idiosyncratic or shared contributions. Intuitively, idiosyncratic contributions should be unique to an individual, which is the definition employed in the current experiment. However, as highlighted above, there might be further scope to distinguish between different aspects of idiosyncratic contributions - for example, regarding different contributions of the interaction and participant-only aspects of personal taste. Future work could, for example, establish whether and how these different aspects of idiosyncratic contributions can be linked to how rewarding voices are perceived to be (e.g. via measuring participant's willingness to listen to the voices – in terms of times spent or money paid). More importantly, perhaps, it is an open question of how universal even shared contributions are. For example, language attitude studies show that there is evidence of ‘shared contributions’ that are specific to a social or cultural group (Bayard et al., 2001; Hendriks, van Meurs, & van Gelder, 2021; Boduch-Grabka & Lev-Ari, 2021; see Sharma et al., 2022 for a recent review). While undoubtedly an example of shared contributions, this example highlights that not all shared contributions are universal but may be culturally or regionally specific.

## 5 Conclusions

In conclusion, we show that listeners both substantially disagree as well as agree on their first impressions of others' voices. Our work reconceptualises vocal impressions as being as much a matter for the listener as the person being heard. This study advances our scientific understanding of this key social phenomenon and given public assumptions around ‘making a good first impression’, our findings also suggest that efforts to change one's own voice will not necessarily affect all listeners the same way. Conversely, and perhaps reassuringly, our findings also suggest that for almost any voice, there is a listener who will positively evaluate it as sounding competent, friendly, or attractive.

## Supplementary Material

Supplementary data to this article can be found online at https://doi.org/10.1016/j.cognition.2024.105881.

Supplementary Material

## Data Availability

All data and analysis scripts are available on the OSF (https://osf.io/ryxvj/?view_only=bbe9fed7f46746a7b088fcd41db92188). All stimulus sets are available from the authors upon request.
