# Supplementary material for: Idiosyncratic and shared contributions shape impressions from voices and faces
Source: Cognition. Author manuscript; Available in PMC 2025 Sep 16. (PMC7618124; doi:10.1016/j.cognition.2024.105881)
Supplement: Supplementary Material [file EMS208279-supplement-Supplementary_Material.docx]

**Supplementary Analysis 1**

Supplementary Table 1 provides a full breakdown of all ICCs components for faces and voices from Experiment 1 and 2.


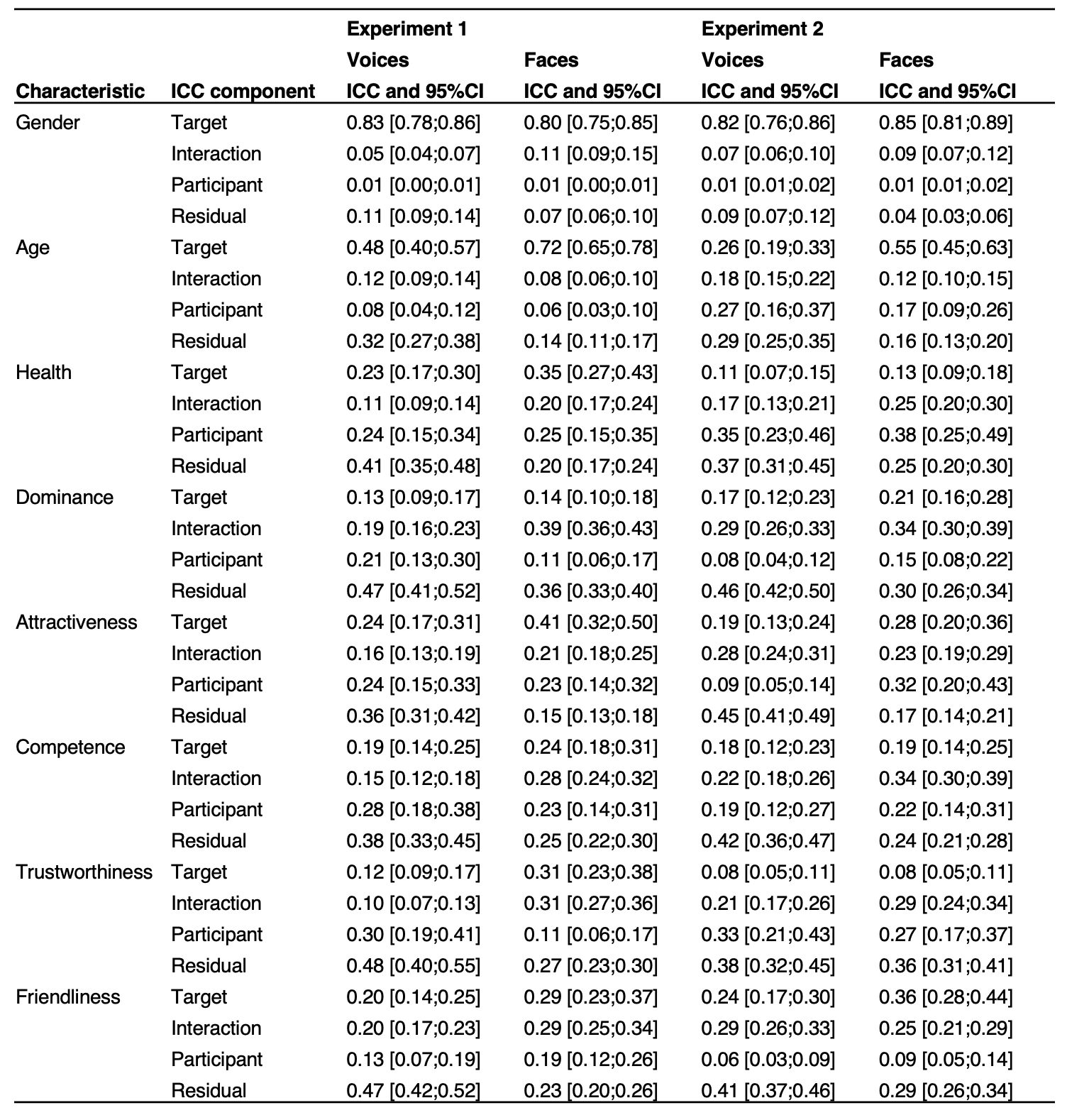


*Supplementary Table 1 Overview of all ICC values and confidence intervals for Experiments 1 and 2.*
